# 

**Published:** 1873-02

**Authors:** 


### TABLE OF CONTENTS.

ORIGINAL COMMUNICATIONS.

A Case of Incapsulated Pleuritic Effusion. By John E. Lockridge, M.D., . 65
Electro-Magnetism in Acute Diseases. By A. Donald, M.D., . . . 71
Mykomyringitis, or Myringitis Parasitica. By A. W. Calhoun, M.D., . . 73
Correspondence. By Charles S. Lewis, M.D., . . . . . . 81

SELECTIONS.

Cervical Adenitis in Children. By Dr. Guersant, . . . . . . 84
The Treatment of Syphilis. By M. P. Ricord, . . . . . . 88
Treatment of Asthma, . . . . . . . . . . . . 90
The Treatment of Amaurosis and Amblyopia by Strychnia, . . . 91
Psoas Abscess—Treatment of, with Sulphurous Acid. By H. Manfred, M.D., 93
On Some of the Most Important Remedies Used in Brain Disease. By William Hammond, M.D., . . . . . . . . . . . . 96

EXTRACTS AND GLEANINGS.

A Good Derivative Liniment—New Theory of Gout, 100; Subclavian Aneurism Cured by Ergotine and Compression, 101; Disease of Ear Attributed to Quinia—Chloral Hydrate in Tetanus Neonatorum—Cathartics in Albuminuria, 102; Wurm on the Diphtheria Question—Calabar Beans for Constipation—Carbolic Acid in Burns, 103; On Cancer of the Sublingual Glands—Good Effects of Aconite in Acute Pneumonia—A Substitute for Quinine, 104; Effects of Various Liquors on the Kidneys and Bladder—Digitalis an Anaphrodisiac—Aconite Poisoning, 105; Conjugal Onanism—Carbolic Acid and Fellow Fever—Maltine—Stramonium an Antidote to Opium Poison, 106; Inhalations of Bromine in Diphtheria and Croup—Hesitation in Curing Eczema—Large Pleuritic Effusion Treated by Thirst—Syphilis, 107; A Simple Method of Arresting Epistaxis—Treatment of Croup—A Singular Way of Opening the Bowels—Chalk Mixture, 108; Varicocele—Consumption—Surgical Treatment of Anasarca—Catarrh, 109; Stuttering Relieved by an Artificial Palate—Management of Obstinate Pruritus—Amaurosis—Cystitis, 110; Iodide of Calcium—Use of Milk in Cancer of Stomach—Gonorrhœa—Delirium Tremens, 111; Morphia Antidotal to Atropia—The Bowel Lesion of Typhoid Fever—Varicose Veins—Pruritus Vulvæ, 112; Palliative Treatment of Ectropion—Diagnostic Sign of Cancer of the Neck of the Womb—Heart Affections—Hiccup—Cure for Corns, 113; Treatment of Asthma—Carbolic Acid in Small Pox—Hemorrhoidal Tumors of the Female Urethra—A Good Local Application in Erysipelas—Chronic Conjunctivitis, 114; Ringworm in Schools—Hydrocele—Treatment of Diabetes Insipidus—Phytolacca in Mammary Inflammations—Good for Chronic Rheumatism, 115; Sick Headache—Cancer—Veratrum Viride an Antidote to Opium—Chronic Orchitis Cured with Phytolacca, 116; Pepsin Wine in Diarrhœa of Young Children—Hare-Lip—Control of Hemorrhage by Ipecac—Catechu and Opium as an Astringent in Gleet—Case of Amaurosis Cured by Electricity, 117; Recurring Tonsillitis—Surgical Affections of Adolescents—Color of the Vagina in Pregnancy—Gelseminum in Irritable Bladder—An Excellent Pill for Dyspepsia and Derangements of the Liver, 118.

EDITORIAL AND MISCELLANEOUS.

The Southern Medical Record; To Our Subscribers, . . . . . . 119
Communications; Correction; Scribner's Monthly, an Illustrated Magazine for the People; Hearth and Home for 1873; Vaccination—Letter from Dr. J. M. Toner, . . . . . . . . . . . . . . 120
List of Payments Received for the Record, Commencing with the January Number, . . . . . . . . . . . . . . 122
Bibliographical, . . . . . . . . . . . . 124

